# Lightweight Deep Learning Model, ConvNeXt-U: An Improved U-Net Network for Extracting Cropland in Complex Landscapes from Gaofen-2 Images

**DOI:** 10.3390/s25010261

**Published:** 2025-01-05

**Authors:** Shukuan Liu, Shi Cao, Xia Lu, Jiqing Peng, Lina Ping, Xiang Fan, Feiyu Teng, Xiangnan Liu

**Affiliations:** 1 School of Information Engineering, China University of Geosciences, Beijing 100083, China; 2104230083@email.cugb.edu.cn (S.L.); 2004230029@email.cugb.edu.cn (F.T.); liuxn@cugb.edu.cn (X.L.); 2 The Second Surveying and Mapping Institute of Hunan Province, Changsha 410004, China; luxia43210231@126.com (X.L.); 15084986337@139.com (J.P.); nana7smile@126.com (L.P.); 15674811345@163.com (X.F.)

**Keywords:** fragmented cropland extraction, ConvNeXt-U, lightweight model, GF-2, remote sensing

## Abstract

Extracting fragmented cropland is essential for effective cropland management and sustainable agricultural development. However, extracting fragmented cropland presents significant challenges due to its irregular and blurred boundaries, as well as the diversity in crop types and distribution. Deep learning methods are widely used for land cover classification. This paper proposes ConvNeXt-U, a lightweight deep learning network that efficiently extracts fragmented cropland while reducing computational requirements and saving costs. ConvNeXt-U retains the U-shaped structure of U-Net but replaces the encoder with a simplified ConvNeXt architecture. The decoder remains unchanged from U-Net, and the lightweight CBAM (Convolutional Block Attention Module) is integrated. This module adaptively adjusts the channel and spatial dimensions of feature maps, emphasizing key features and suppressing redundant information, which enhances the capture of edge features and improves extraction accuracy. The case study area is Hengyang County, Hunan Province, China, using GF-2 remote sensing imagery. The results show that ConvNeXt-U outperforms existing methods, such as Swin Transformer (Acc = 85.1%, IoU = 79.1%), MobileNetV3 (Acc = 83.4%, IoU = 77.6%), VGG16 (Acc = 80.5%, IoU = 74.6%), and ResUnet (Acc = 81.8%, IoU = 76.1%), achieving an IoU of 79.5% and Acc of 85.2%. Under the same conditions, ConvNeXt-U has a faster inference speed of 37 images/s, compared to 28 images/s for Swin Transformer, 35 images/s for MobileNetV3, and 0.43 and 0.44 images/s for VGG16 and ResUnet, respectively. Moreover, ConvNeXt-U outperforms other methods in processing the boundaries of fragmented cropland, producing clearer and more complete boundaries. The results indicate that the ConvNeXt and CBAM modules significantly enhance the accuracy of fragmented cropland extraction. ConvNeXt-U is also an effective method for extracting fragmented cropland from remote sensing imagery.

## 1. Introduction

Hilly terrain has resulted in the formation of fragmented cropland, which consists of small, discontinuous plots with fragmented usage rights. Crops on these lands also show fragmented characteristics: they are small in area, diverse in type, and discontinuous in distribution. The boundaries of fragmented cropland are irregular and unclear, often interspersed with ponds, small woodlands, and buildings, complicating their extraction and presenting significant challenges. Historically, extracting fragmented cropland has often faced issues like inaccurate boundary delineation, misidentification of other land covers as cropland, or misclassification of cropland as other land covers [1].

Cropland fragmentation in China significantly affects agricultural productivity and land use efficiency. Fragmented cropland lacks potential for large-scale cultivation and primarily supports small-scale farming, contributing only 8% to total crop yield [2,3]. Additionally, rural aging and climate change have increased abandonment rates of fragmented cropland, posing a substantial threat to the sustainable development of Chinese agriculture [4,5]. Different ecological regions require appropriate crop distribution and management for fragmented cropland [6]. Therefore, high-precision extraction of fragmented cropland is crucial for its effective management and optimization.

With advancements in remote sensing technology, image resolution has steadily improved, evolving from early 500 m resolution [7] to current meter- and centimeter-level resolutions [8,9,10]. Despite these advancements, research on cropland extraction using meter- to sub-meter resolution imagery remains limited, especially for large-scale monitoring in the complex terrain of southern China. High-resolution imagery provides enhanced spatial detail and contextual features, improving information accuracy but also introducing challenges. Spectral variability (i.e., the same object exhibiting different spectral characteristics under varying conditions) and spectral similarity (i.e., different objects exhibiting similar spectral characteristics under the same conditions) continue to significantly impact the effectiveness of high-resolution imagery applications. Traditional classification algorithms, such as decision trees [11,12], support vector machines [13,14,15], artificial neural networks [16], and random forests [12,15,17], often heavily rely on spectral information for feature extraction, leading to significant limitations in complex cropland environments. These methods mainly focus on low-level features while neglecting higher-level semantic information, resulting in severe salt-and-pepper noise in classification results, particularly in fragmented and undulating cropland areas. Although these traditional methods have been widely used, they are labor-intensive, costly, time-consuming, and have limited automation. Moreover, the diversity of high-resolution sensors, variability in imaging environments, complexity of scene targets, and the dispersed nature of cropland changes add further challenges to traditional classification algorithms. As remote sensing imagery resolution continues to increase, traditional methods struggle to adapt to new sensors and manage large volumes of high-resolution data [18]. Consequently, there is significant hope in academia and industry that deep learning technologies will overcome the limitations of existing methods.

In recent years, advancements in remote sensing and deep learning technologies have increased the use of automated learning and complex feature extraction in remote sensing image processing. Deep learning models, including Convolutional Neural Networks (CNNs) [19,20,21], Fully Convolutional Networks (FCNs) [22,23,24], Recurrent Neural Networks (RNNs) [25,26,27,28], and Generative Adversarial Networks (GANs) [29,30], have shown greater flexibility and adaptability than traditional methods. These models automatically extract complex features from large-scale data through deep network training, leading to more accurate identification of fragmented cropland and other land cover types. Advances in remote sensing technology have expanded data resolution from moderate (10–30 m) [31,32,33,34] to ultra-high resolution (better than 1 m) [29,35,36], significantly improving the precision of land cover classification. In cropland identification, deep learning models are classified into single-task, multi-task [37], and segmentation and classification models, each suited for specific recognition tasks. Recent research has explored novel methods integrating large-scale AI models with remote sensing imagery, such as the Segment Anything Model (SAM) [38], which has significantly improved the efficiency and accuracy of cropland identification [39]. These technological advancements offer new opportunities and challenges for remote sensing image processing and land cover classification.

Existing research on cropland extraction using deep learning methods has predominantly focused on edge-based recognition [9,40,41,42]. This approach works well for large, regular croplands with clear boundaries but is less effective for fragmented croplands with irregular and unclear boundaries. Additionally, deep learning methods for delineating cropland boundaries often face problems like open or incomplete boundaries [9]. Due to limitations in remote sensing image resolution and the characteristics of fragmented cropland, current deep learning methods for boundary delineation are unsuitable for extracting fragmented cropland. Consequently, we focused on pixel-based approaches for cropland extraction [43]. As deep learning models become more complex and remote sensing data volumes increase, these methods face challenges related to high computational demands and large-scale data processing, requiring substantial computational resources and storage space. This limitation hinders their widespread application. Therefore, we have developed lightweight solutions to significantly reduce computational resource requirements while maintaining model performance. This is especially critical in cropland extraction, where efficient models can process large volumes of remote sensing data more quickly. In this context, we propose ConvNeXt-U, a model optimized from the ConvNeXt architecture specifically for high-precision cropland boundary identification. The novelty of our research is demonstrated by the following aspects:

(1) We have developed ConvNeXt-U, a lightweight model that reduces computational resource demands and is suitable for deployment in resource-constrained environments.
(2) The model includes an attention mechanism that automatically focuses on key regions in images, enhancing feature extraction and improving the accuracy of boundary and detail recognition in cropland.
(3) Our model maintains high computational efficiency, making it suitable for processing large-scale remote sensing data and enhancing its applicability in agricultural management.

The structure of this paper is as follows: Section 2 provides an overview of the research area and datasets. Section 3 details the ConvNeXt-U model’s design. Section 4 presents the experimental results and analysis, Section 5 discusses the findings, and Section 6 summarizes the results and offers suggestions for future research.

## 2. Study Area and Data

### 2.1. Study Area

**Figure 1:**
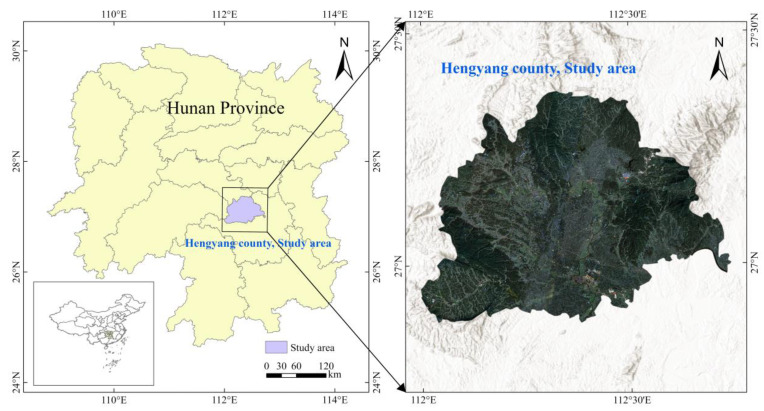
Map of the study area in Hengyang County, Hunan Province, China.

The study area is Hengyang County (26°58′11″ N, 112°22′14″ E), located in central Hunan Province, China (Figure 1). It covers 2558.61 square kilometers, with about 590.67 square kilometers dedicated to cropland. The terrain is mostly hilly and mountainous, with higher elevations in the south and lower elevations in the north, leading to varied hydrological conditions and land use patterns. Hengyang County experiences a subtropical monsoon climate with distinct seasons and ample rainfall. The average annual temperature is approximately 17 °C, and the average annual precipitation is around 1300 mm, providing favorable conditions for crop growth. The main land cover types are forests, shrublands, cropland, and water bodies. Cropland, mainly consisting of rice paddies and dry fields, is irregular and fragmented due to topographical constraints and traditional farming practices. The primary crops are rice, wheat, corn, and soybeans, with rice being the staple crop. Rice paddies are primarily found in the lower-lying areas, benefiting from abundant water resources for irrigation.

**Figure 2:**
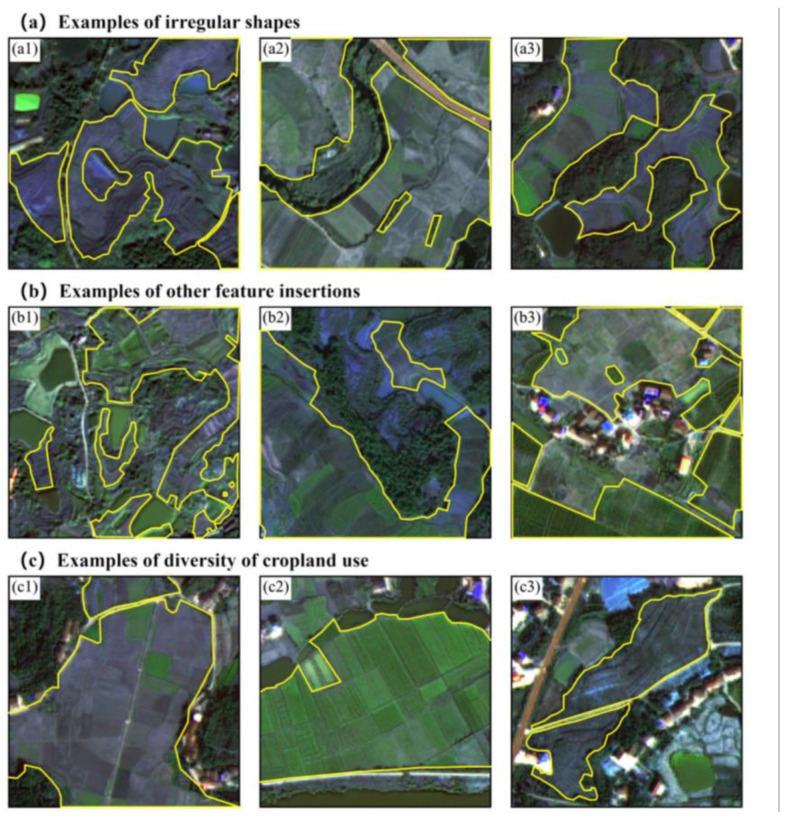
The complexity of cropland in Hengyang County is illustrated in the following figures. Subfigures (**a1**–**a3**) illustrate the irregularity of cropland in the region. Subfigures (**b1**–**b3**) demonstrate the complexity of cropland interspersed with other land features in the region. Subfigures (**c1**–**c3**) demonstrate the diversity of cropland use in the region, including areas interspersed with fallow and non-fallow land. Yellow circles highlight the cropland areas.

Cropland in Hengyang County exemplifies fragmentation significantly influenced by topography and climate. As shown in Figure 2, the cropland features numerous small, irregular plots interspersed with residential areas, ponds, forests, and roads. Boundaries between cropland and hilly areas are often indistinct. Cropland use in Hengyang County is irregular, with various crops spread across numerous plots of different sizes and some land left fallow. Fallow and non-fallow areas are interspersed, adding to the complexity of land use patterns. This complex cropland landscape poses significant challenges for large-scale cropland extraction in the region.

### 2.2. Gaofen-2 Satellite Image

We collected GF-2 satellite images of Hengnan County, taken during the 2023 crop harvest period, highlighting the region’s diverse cropland landscape. The GF-2 satellite, launched by China, offers high-resolution imagery with a 0.8 m panchromatic resolution and a 3.2 m multi-spectral resolution. Details of the GF-2 imagery parameters are provided in Table 1. This high resolution makes the imagery applicable to various fields, including agriculture, forestry, urban planning, environmental protection, and land resource management.

In this study, we processed GF-2 satellite images to improve their spatial resolution to 1 m. This enhancement improves image detail, enabling clearer identification of surface features and finer details, thereby providing more precise data for various applications. High-resolution data are particularly suited for small-scale plot analysis and detailed land use studies, improving the accuracy of various analyses and decisions. Using GF-2 images allows us to better capture the characteristics of fragmented cropland, thus improving the accuracy of its extraction.

The ground truth for fragmented cropland in the GF-2 dataset was manually delineated. To ensure effective model training and validation, the samples were randomly split into training and validation sets with an 8:2 ratio, with no overlap between them. In the following sections, we refer to this dataset as the Hengyang dataset. We also collected a cropland dataset from Denmark. The Denmark dataset is publicly accessible through the European Union Land Parcel Identification System (LPIS) at: https://collections.eurodatacube.com/, accessed on 13 October 2024. The dataset consists of two cloud-free Sentinel-2 images from 8 May 2016, with a true-color composite at a 10 m resolution [44].

## 3. Methods

**Figure 3:**
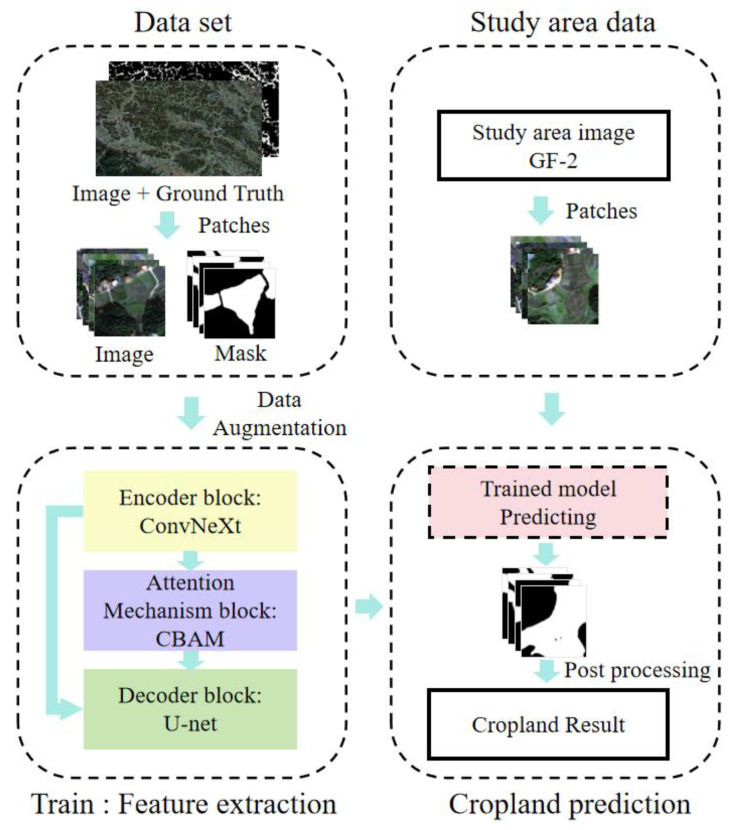
The workflow for extracting fragmented cropland using GF-2 remote sensing imagery includes several key components. The ConvNeXt encoder extracts and compresses features from the raw image. The U-Net decoder reconstructs images using these features. The CBAM attention mechanism enhances accuracy and robustness by emphasizing key regions and information. Integrating these three components significantly improves the effectiveness of cropland fragmentation extraction from remote sensing imagery.

Figure 3 illustrates the workflow for extracting fragmented cropland using Gaofen-2 remote sensing imagery. Initially, the images undergo preprocessing, including orthorectification and pansharpening. Subsequently, we utilize a neural network, ConvNeXt-U, which comprises an encoder, a decoder, and an attention mechanism. The encoder, constructed from ConvNeXt modules, efficiently extracts multi-scale features of the fragmented cropland, providing detailed and accurate information for decoding. The decoder, based on U-Net, restores spatial resolution, integrates feature information, and applies the attention mechanism to reconstruct the image and extract key features, ensuring high-quality extraction. The attention mechanism, CBAM (Convolutional Block Attention Module), improves the model’s feature representation, leading to more accurate recognition and segmentation of cropland areas, particularly in complex terrains and detailed scenarios.

### 3.1. ConvNeXt

ConvNeXt, introduced by Facebook AI Research (FAIR) in 2022, is a contemporary Convolutional Neural Network architecture designed as an alternative to the prevalent Transformer models. Based primarily on the classic ResNet architecture [45], ConvNeXt includes several design improvements aimed at enhancing model performance and efficiency. Initially, the developers employed training strategies akin to those used for vision Transformers to train ResNet, significantly boosting the performance of ResNet-50. They then introduced five key design optimizations:

(1) Macroscopic Design: Inspired by the multi-stage architecture of Swin Transformers, the researchers adjusted feature map resolutions and computational resources at various stages to enhance the network’s expressive capacity.
(2) ResNeXt Concept: Grouped convolution techniques enable the sharing of filters across multiple groups, thereby increasing the network’s width without significantly raising computational demands.
(3) Inverted Bottleneck: By adopting an inverted bottleneck structure akin to that used in Transformers, the model significantly increased the hidden dimensions of the multi-layer perceptron (MLP) blocks relative to the input dimensions, thereby reducing computational costs.
(4) Large Kernel Sizes: The architecture benefits from larger convolutional kernel sizes (e.g., from 3 × 3 to 7 × 7), enhancing performance while keeping computational complexity manageable.
(5) Layered Microscopic Design: At the microscopic level, several configurations were optimized: ReLU [46] activation functions were replaced with GELU [47], the number of activation and normalization layers was reduced, and layer normalization was introduced instead of batch normalization.

**Figure 4:**
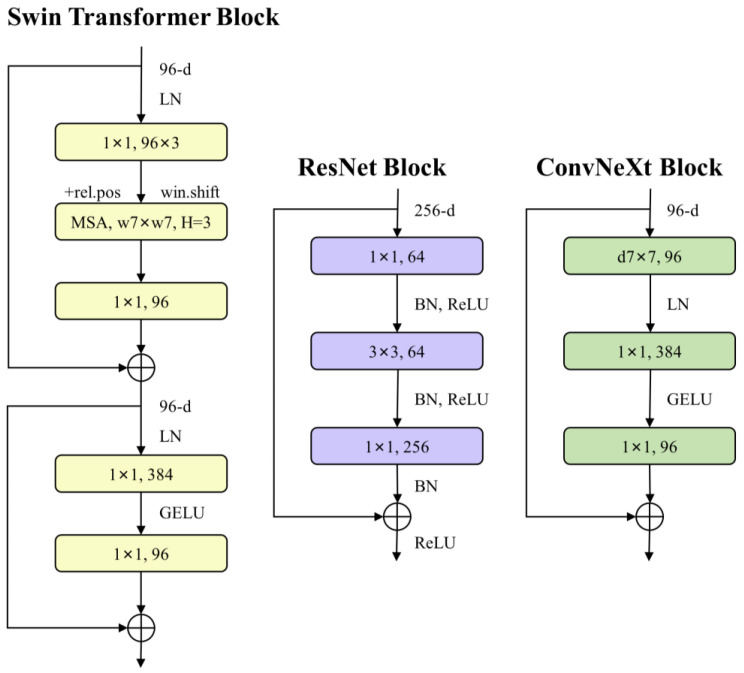
Comparison of Swin Transformer, ResNet, and ConvNeXt block structures.

These optimizations enable ConvNeXt to achieve faster inference and higher accuracy than the Swin Transformer, while maintaining similar FLOPs. Specifically, ConvNeXt’s throughput is approximately 49% higher than that of the Swin Transformer at similar FLOPs [48]. ConvNeXt was selected for its lightweight architecture, which achieves performance on par with larger models. Figure 4 compares the ConvNeXt module design with that of the reference model. This architecture underscores the ongoing significance of Convolutional Neural Networks in modern deep learning and offers new directions for future model development.

### 3.2. ConvNeXt-U

**Figure 5:**
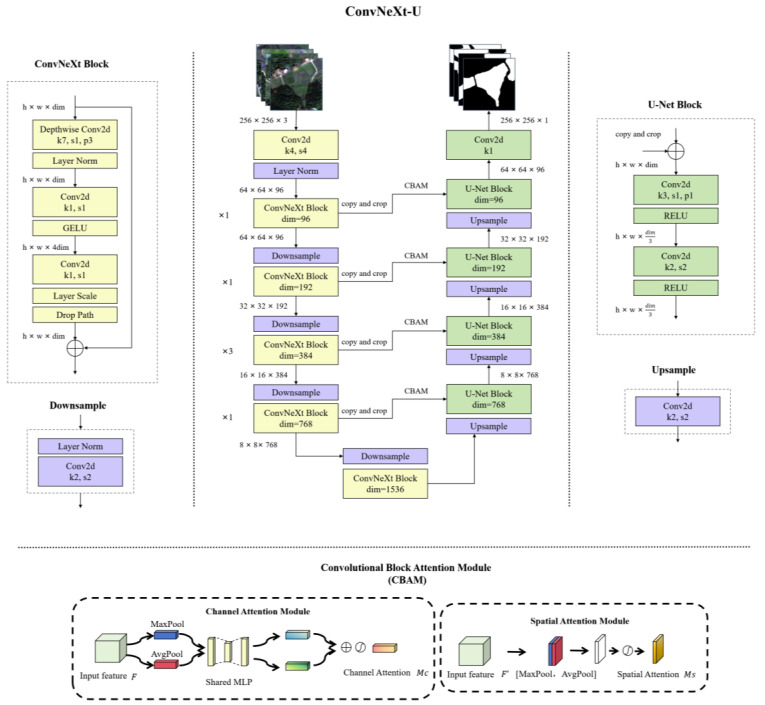
The proposed ConvNeXt-U architecture comprises three main components: the ConvNeXt encoder, the U-Net decoder, and the CBAM attention mechanism.

This study introduces an enhanced U-Net network structure, termed ConvNeXt-U (Figure 5), which is optimized for the efficient extraction of fragmented cropland features from remote sensing images. ConvNeXt-U incorporates the ConvNeXt module as the encoder, utilizes the classic symmetric decoder structure of U-Net, and employs the CBAM (Convolutional Block Attention Module) for attention mechanisms.

ConvNeXt-U preserves the U-Net’s U-shaped structure while replacing the encoder with ConvNeXt modules. These modules utilize large convolutional kernels (e.g., 7 × 7) and stacked blocks (1:1:3:1) to expand the receptive field and capture both large-scale and local features. The initial layer comprises 96 channels, and the activation function for the ConvNeXt blocks is GELU. The decoder retains the symmetric structure of U-Net, restoring spatial resolution through up-sampling and convolutional layers, with RELU serving as the activation function. Each stage of the decoder receives skip connection features from the encoder, with the Convolutional Block Attention Module (CBAM) [49] applied to these connections. The features are up-sampled to the original resolution using transposed convolutions. CBAM includes channel and spatial attention mechanisms, which enhance feature representation in both dimensions, as illustrated in Figure 5. Channel attention generates channel weights via global average and max pooling, followed by a fully connected layer and a Sigmoid function. Spatial attention, applied after channel attention, pools feature maps in the channel dimension using max and average pooling, and derives spatial weights through convolutional layers.

By incorporating ConvNeXt modules, ConvNeXt-U integrates multi-scale information during feature extraction, which enhances its ability to capture complex terrain features, including subtle boundaries and internal structures in fragmented cropland. The CBAM attention mechanism further enhances feature selection, aiding the model in suppressing background noise and focusing on critical terrain features. Compared to the traditional U-Net, ConvNeXt-U demonstrates significant improvements in Accuracy, boundary detection, and detail preservation.

### 3.3. Evaluation Metric

We evaluated the classification performance of each model using five common metrics: Accuracy (ACC), Precision, Recall, F1 Score (F1), and Intersection over Union (IoU). We also visualized the classification maps from different models to enable qualitative comparisons.

Accuracy (Acc) is a fundamental metric for evaluating model performance. It represents the ratio of correctly predicted instances to the total number of predictions. In this context, it measures the ratio of correctly identified cropland to the total number of land objects. The formula is as follows:

(1) $$Acc=\frac{TP+TN}{TP+TN+FN+FP}$$

Accuracy is a reliable metric only when the dataset is balanced, with nearly equal values for false positives and false negatives. Thus, other parameters must be considered to comprehensively assess the model’s performance.

Precision is the ratio of true positive samples to all samples classified as positive. Specifically, it measures the ratio of correctly identified cropland to the total amount of extracted cropland. The formula is as follows:

(2) $$Precision=\frac{TP}{TP+FP}$$

Recall measures the proportion of true positive samples correctly identified by the model. Specifically, it represents the probability that a sample is predicted as positive given that it is actually positive, indicating the likelihood that the model correctly identifies cropland among all actual cropland areas. The formula for Recall is as follows:

(3) $$Recall=\frac{TP}{TP+FN}$$

The F1 score (F1) is the harmonic mean of Precision and Recall, commonly used in binary classification problems. It accounts for both false positives and false negatives, making it more suitable than Accuracy, particularly when class distribution is imbalanced. In cropland extraction, the F1 score evaluates the model’s overall performance in identifying both cropland and non-cropland areas. The formula for the F1 score is as follows:

(4) $$F1=2\times \frac{Precision\times Recall}{Precision+Recall}$$

The Intersection over Union (IoU) is a key metric for assessing the performance of object detection and image segmentation algorithms. IoU quantifies the overlap between predicted and actual results by calculating the ratio of their intersection to their union. It measures how well the model’s predicted cropland areas overlap with the actual cropland areas. The formula for IoU is as follows:

(5) $$IoU=\frac{TP}{TP+FP+FN}$$

where, TP, TN, FP, and FN represent true positives, true negatives, false positives, and false negatives, respectively. TP and TN refer to the number of pixels correctly identified as agricultural and non-agricultural parcels, respectively, while FN and FP refer to the number of pixels incorrectly identified as non-agricultural (i.e., omissions) and agricultural parcels (i.e., mistakes).

### 3.4. Implementation Details

To analyze the satellite and ground truth images from our study area, we cropped them into non-overlapping 256 × 256 pixel patches. To enhance the ConvNeXt-U model’s generalization, we applied data augmentation techniques such as horizontal and vertical flipping, mixed sample generation, and color jittering during preprocessing. These methods aimed to increase the model’s robustness and performance under varying conditions. To reduce the proportion of negative samples, we filtered the images, retaining only those with at least 20% agricultural pixels. This process was also applied to the Denmark dataset. This process resulted in 4152 fragmented cropland samples from the training and validation areas. We used the Stochastic Gradient Descent (SGD) optimizer with an initial learning rate of 0.001 and a weight decay of 10^−4^ during model training for edge prediction. These parameters optimized the learning process and ensured optimal model performance. We also implemented the weight initialization strategy proposed by Liu et al. (2019) [50] to ensure effective parameter initialization and enhance model performance. We followed recommended loss functions and training protocols from existing research to train other models, including Swin Transformer, MobileNetV3, ResNet, and VGG16 in our experiments. The models were trained on an NVIDIA GeForce RTX 4070 GPU for 100 epochs with a batch size of 4. These settings and procedures aim to achieve high model accuracy and satisfactory results in practical applications.

## 4. Results

### 4.1. Ablation Study for the Module

We performed an ablation study to evaluate the effectiveness of the proposed ConvNeXt module. Specifically, we replaced the encoder of the ConvNeXt-U model with the encoder from the original U-Net model and evaluated both models on the Hengyang and Denmark datasets to compare their performance across different contexts. Table 2 presents the accuracy of the ConvNeXt-U and U-Net models on both datasets. On the Hengyang dataset, the U-Net model achieved an IoU of 75.6% for fragmented cropland extraction, whereas the ConvNeXt-U model, incorporating the ConvNeXt module, reached an IoU of 79.0%, showing a 3.4% improvement. On the Denmark dataset, the U-Net model obtained an IoU of 84.8%, while the ConvNeXt-U model achieved an IoU of 86.8%, reflecting a 2.0% increase. These results demonstrate that the ConvNeXt module substantially enhances extraction accuracy across various regions and datasets, thereby confirming its effectiveness.

Furthermore, we conducted an evaluation to assess the effectiveness of the Convolutional Block Attention Module (CBAM). Specifically, we incorporated the CBAM module into both ConvNeXt-U and U-Net models, yielding the ConvNeXt-U (+CBAM) and U-Net (+CBAM) versions. These models were evaluated on the Hengyang and Denmark datasets to assess the impact of the CBAM module across different network architectures. Table 3 and Table 4 show that the U-Net (+CBAM) model achieved an IoU of 77.9% for fragmented cropland extraction on the Hengyang dataset, representing a 2.3% improvement over the original U-Net model (IoU = 75.6%). On the Denmark dataset, the U-Net (+CBAM) model achieved an IoU of 86.5%, reflecting a 1.7% increase over the original U-Net model (IoU = 84.8%).

In contrast, the addition of the CBAM module led to only a modest performance improvement in the ConvNeXt-U model. On the Hengyang dataset, the ConvNeXt-U model with CBAM exhibited a 0.5% improvement compared to the version without CBAM. On the Denmark dataset, the improvement was even more modest, at only 0.2%. The slight improvements across both datasets can be attributed to ConvNeXt’s strong feature representation capabilities, which already produce sufficiently robust features, leaving little room for further enhancement by the CBAM module. Additionally, the CBAM mechanism may be less compatible with the ConvNeXt architecture, reducing its effectiveness in enhancing performance. Despite these modest improvements, incorporating the CBAM module into the ConvNeXt-U model still led to a slight optimization in performance. The comprehensive experimental results show that our proposed model performs well on both the Hengyang and Denmark datasets, suggesting that it is adaptable to different regions and demonstrates strong generalization capabilities.

**Figure 6:**
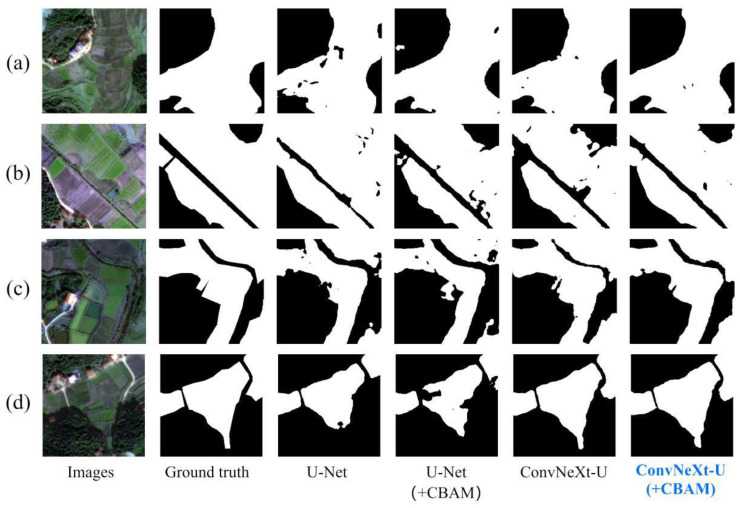
Comparison of fragmented cropland extraction results using U-Net, ConvNeXt-U, and ConvNeXt-U with the CBAM attention mechanism. Panels (**a**–**d**) show typical examples for each of the four methods.

Additionally, Figure 6 shows visual results of fragmented cropland extraction in Hengyang County, using both the ConvNeXt-U and U-Net models, with and without the CBAM module. The results demonstrate that the ConvNeXt module reduced undetected cropland, emphasizing its role in enhancing cropland recognition and extraction accuracy. This ablation study concludes that the ConvNeXt module significantly improves the accuracy and reliability of fragmented cropland extraction, offering valuable insights for future research and applications.

Figure 6 further illustrates the visual results of fragmented cropland extraction in Hengyang County using the ConvNeXt-U and U-Net models, both with and without the CBAM module. The comparison shows that adding the CBAM module significantly reduced undetected cropland. Specifically, the ConvNeXt-U model with the CBAM module showed fewer undetected cropland instances, suggesting that the CBAM module enhanced the model’s recognition and extraction capabilities, thereby maintaining higher accuracy and reliability in complex environments. The ConvNeXt-U (+CBAM) model was employed to extract fragmented cropland in the study area.

### 4.2. Extraction of Fragmented Cropland Fragmentation on Study Area Gaofen-2 Images

We evaluated the performance of the ConvNeXt-U model, Swin Transformer, MobileNetV3, ResUnet, and VGG16 in fragmented cropland extraction using five metrics: Accuracy (Acc), Precision, Recall, F1 score, and Intersection over Union (IoU). We also analyzed their parameter counts, FLOPs, and throughput. Table 5 demonstrates that ConvNeXt-U (+CBAM) outperformed the other models in fragmented cropland extraction, achieving the best results. ConvNeXt-U (+CBAM) achieved the highest scores in both Acc and IoU, with values of 85.2% and 79.5%, respectively. Notably, ConvNeXt-U (+CBAM) outperformed MobileNetV3 (IoU = 77.6%), ResUnet (IoU = 76.1%), and VGG16 (IoU = 74.6%) by 1.9%, 3.4%, and 4.9%, respectively, in terms of IoU. ConvNeXt-U (+CBAM) demonstrated performance comparable to that of the Swin Transformer (Acc = 85.1%, IoU = 79.1%) in fragmented cropland extraction. Although ConvNeXt-U (+CBAM) outperformed the Swin Transformer by only 0.1% in Acc and 0.4% in IoU, as shown in Table 6, it surpassed the Swin Transformer in parameter count, FLOPs, and throughput. ConvNeXt-U (+CBAM) had fewer FLOPs and parameters, yet higher throughput. Table 5 and Table 6 further show that although the throughput of MobileNetV3 was only two units lower than that of ConvNeXt-U (+CBAM); its IoU was 1.9% lower. Table 6 also shows that ResUnet and VGG16 performed worse than ConvNeXt-U (+CBAM) across all three metrics: parameter count, FLOPs, and throughput.

**Figure 7:**
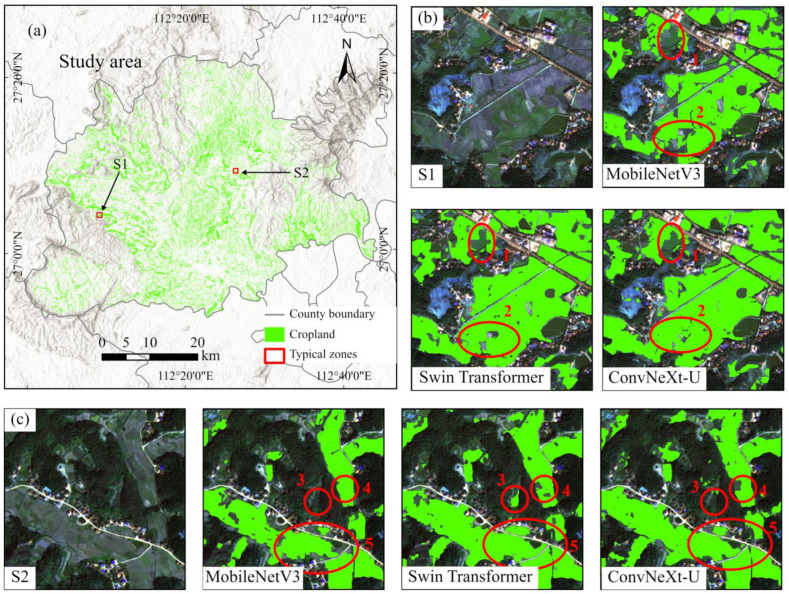
Results of fragmented cropland extraction in the study area using MobileNetV3, Swin Transformer, and ConvNeXt-U models. (**a**) Extraction results for the ConvNeXt-U model. (**b**,**c**) Typical croplands marked as S1 and S2, shown with the original true-color GF-2 imagery, and results from MobileNetV3, Swin Transformer, and ConvNeXt-U. Red circles numbered 1 to 5 highlight performance variations among the three models.

Figure 7 presents the final results of fragmented cropland extraction in the study area using the ConvNeXt-U (+CBAM), Swin Transformer, and MobileNetV3 models, along with several representative landscape subsets. Specifically, Figure 7a displays the results of fragmented cropland extraction with the ConvNeXt-U (+CBAM) model. Figure 7b offers a detailed comparison of the study area, including the original image and corresponding extraction results, labeled as S1 and S2. Red circles highlight regions where the models show notable performance differences. Notably, circles 1 and 5 highlight that the ConvNeXt-U (+CBAM) model extracted fragmented cropland boundaries more clearly and completely. In contrast, circles 2 to 4 reveal that the Swin Transformer and MobileNetV3 models occasionally misclassified non-cropland as cropland or vice versa. Therefore, the ConvNeXt-U (+CBAM) model exhibited superior extraction accuracy for fragmented cropland over both Swin Transformer and MobileNetV3.

**Figure 8:**
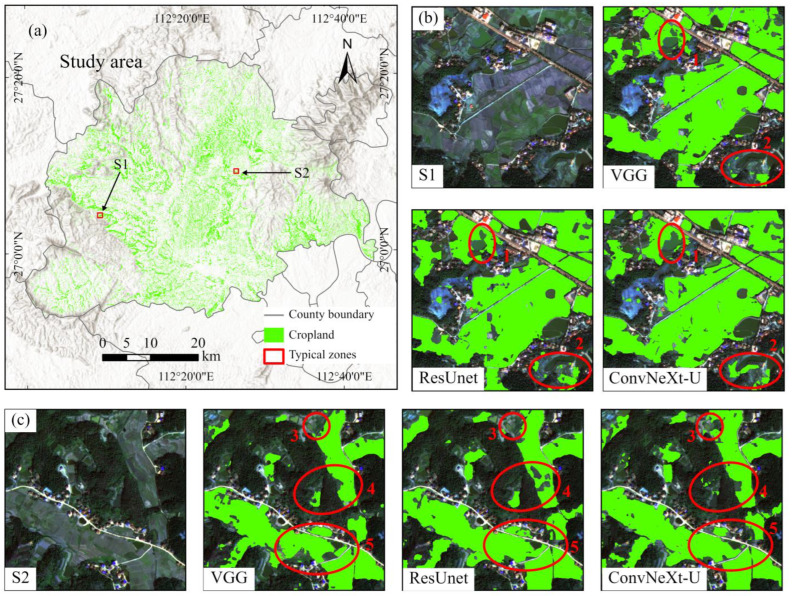
Results of fragmented cropland extraction in the study area using VGG16, ResUnet, and ConvNeXt-U models. (**a**) Extraction results for the ConvNeXt-U model. (**b**,**c**) Typical croplands marked as S1 and S2, shown with the original true-color GF-2 imagery, and results from VGG16, ResUnet, and ConvNeXt-U. Red circles numbered 1 to 5 highlight performance variations among the three models.

Figure 8 shows the final extraction results for fragmented cropland in the study area using the ConvNeXt-U (+CBAM), ResUnet, and VGG16 models, along with several representative landscape subsets. ConvNeXt-U (+CBAM) performed especially well in two highlighted regions (circles 2 and 3). This model more accurately identified smaller cropland patches, reducing the occurrence of missed cropland. Circle 4, located in the center of S2, shows that ConvNeXt-U (+CBAM) made fewer errors in misclassifying non-cropland as cropland compared to VGG16 and ResUnet. Additionally, circles 1 and 5 indicate that ConvNeXt-U (+CBAM) outperformed ResUnet and VGG16 in delineating the boundaries of fragmented cropland. Combined results from Figure 7 and Figure 8 clearly demonstrate that ConvNeXt-U (+CBAM) outperformed the other models in extracting fragmented cropland.

### 4.3. Map of Fragmented Cropland

**Figure 9:**
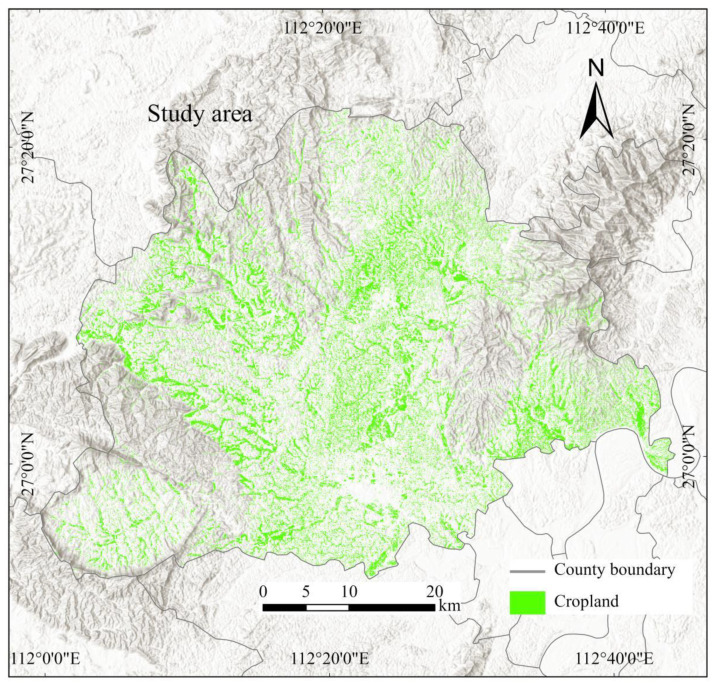
Results of fragmented cropland extraction in the study area using VGG16, ResUnet, and ConvNeXt-U models. Final extraction results for the study area using the ConvNeXt-U model are shown.

Figure 9 illustrates cropland fragmentation in the study area for 2023. The eastern part of the study area, dominated by the Hengshan Mountains, has limited cropland distribution. Mountainous terrain in the western and northern regions concentrates cropland in the central area, which features abundant water resources and relatively flat terrain. Despite fragmentation, these flat areas are suitable for crop cultivation, particularly rice. Cropland near rivers is generally more continuous, less fragmented, and larger. In contrast, cropland near residential areas is more fragmented, exhibits higher spatial heterogeneity, and includes a greater mix of features like ponds and buildings, leading to more diverse crops.

## 5. Discussion

In this study, the proposed ConvNeXt-U model features a simple structure while achieving excellent performance in fragmented cropland extraction. We selected U-Net as the base network for its simplicity and efficiency, which ensures high performance. We incorporated the ConvNeXt module, which employs a pure Convolutional Neural Network (CNN) architecture as its encoder. Compared to Transformer models, ConvNeXt provides a more streamlined and lightweight structure, with comparable performance. The CBAM attention mechanism integrates seamlessly into existing CNN architectures without significantly increasing computational complexity or model parameters. We selected U-Net as the base network for its simplicity and efficiency, which ensure excellent performance. In 2023, the ConvNeXt team introduced an improved version, ConvNeXt V2 [51]. This version enhances the original model by incorporating a Fully Convolutional Masked Autoencoder (FCMAE) and Global Response Normalization (GRN). The FCMAE framework masks random parts of the input image, requiring the model to recover these areas, which compels the model to learn both global and local features, thereby enhancing its generalization capability. The GRN layer, a novel CNN layer, normalizes feature maps across channels, improving feature competition and addressing the feature collapse problem observed in ConvNeXt V1, which had redundant activations (dead or saturated neurons) between channels. Future work may involve replacing ConvNeXt V1 with ConvNeXt V2 to enhance the accuracy of fragmented cropland extraction. Additionally, while the CBAM attention mechanism focuses on local context, future research could explore global attention mechanisms to better capture global dependencies.

Our method has demonstrated strong performance and efficiency in extracting fragmented cropland using GF-2 imagery. However, GF-2 imagery has a maximum spatial resolution of 0.8 m and limited spectral resolution, with only four bands plus a panchromatic band. These spatial and spectral resolution constraints limit the ability to capture small or blurred cropland patches. Future research should explore using higher spatial resolution remote sensing imagery or UAV images to achieve more accurate extraction of fragmented cropland [52,53]. Additionally, the current imagery’s spatial and spectral resolution limits the differentiation of various crops. Higher resolution imagery could address this limitation.

In this study, the samples used to train our model and other existing methods were manually collected. Sample collection is still a labor-intensive process for deep neural networks. Future research could explore automated labeling methods, such as Canny edge detection or Generative Adversarial Networks (GANs), to reduce manual labeling costs and improve the applicability of our proposed method [40,54].

Our proposed network is also applicable to village extraction tasks, which involve dispersed, fragmented, and irregular boundaries. Despite its simplicity, our network model is highly effective, providing efficient computation and low latency, making it ideal for real-time processing tasks. With the ongoing advancements in computational resources and hardware, these models are expected to find broader applications in mobile computing and real-time processing.

## 6. Conclusions

In this study, we propose a novel automated method for extracting fragmented cropland by enhancing the U-Net network to better capture cropland edges and spectral features. The enhanced network model is referred to as ConvNeXt-U (+CBAM). We evaluated the performance of this method using data from Hengyang County, Hunan Province. The ConvNeXt-U (+CBAM) model, trained on the Hengyang County cropland dataset, achieved an Accuracy (Acc) of 85.2% and an Intersection over Union (IoU) of 79.5% for fragmented cropland extraction. We performed ablation experiments on the improved model and compared its performance with that of the Swin Transformer, MobileNetV3, ResUnet, and VGG16 models.

(1) Replacing the U-Net encoder with ConvNeXt improves the model’s ability to extract fragmented cropland from remote sensing images, resulting in higher extraction accuracy compared to the original U-Net.
(2) We incorporated the CBAM attention mechanism into both the U-Net and the enhanced ConvNeXt-U models. Comparative experiments consistently showed that CBAM-augmented models achieved superior extraction accuracy compared to their baseline counterparts. This indicates that the CBAM mechanism improves the model’s ability to capture detailed features of fragmented cropland, resulting in more accurate extraction.
(3) A comparison of the Swin Transformer, MobileNetV3, ResUnet, and VGG16 models reveals that the enhanced ConvNeXt-U (+CBAM) model outperforms them in fragmented cropland extraction.

ConvNeXt-U (+CBAM) effectively extracts cropland of different shapes and sizes from high-resolution remote sensing images. This method performs well even outside the training region, providing a promising solution for fragmented cropland extraction across various mapping scales.

## Figures and Tables

**Table 1:** Characteristics of multi-spectral Gaofen-2 images used in the study.

Gaofen-2 Bands	Wavelength (nm)	Resolution (m)
1 (blue)	450–520	3.2
2 (green)	520–590	3.2
3 (red)	630–690	3.2
4 (NIR)	760–900	3.2
5 (panchromatic)	450–900	0.8

**Table 2:** Results of the ConvNeXt module ablation study.

		Acc (%)	Precision (%)	Recall	F1	IoU (%)
Hengyang Dataset	ConvNeXt-U	84.6	79.5	0.992	0.883	79.0
	U-Net	81.3	76.1	0.991	0.861	75.6
Denmark Dataset	ConvNeXt-U	90.8	92.1	0.938	0.929	86.8
	U-Net	89.4	86.0	0.951	0.922	84.8

**Table 3:** Results of the CBAM module ablation study on the Hengyang dataset.

	Acc (%)	Precision (%)	Recall	F1	IoU (%)
ConvNeXt_U (+CBAM)	85.2	80.5	0.986	0.886	79.5
ConvNeXt_U	84.6	79.5	0.992	0.883	79.0
U-Net (+CBAM)	83.6	78.7	0.987	0.876	77.9
U-Net	81.3	76.1	0.991	0.861	75.6

**Table 4:** Results of the CBAM module ablation study on the Denmark dataset.

	Acc (%)	Precision (%)	Recall	F1	IoU (%)
ConvNeXt_U (+CBAM)	91.1	91.2	0.957	0.930	87.0
ConvNeXt_U	90.8	92.1	0.938	0.929	86.8
U-Net (+CBAM)	89.8	88.0	0.937	0.925	86.5
U-Net	89.4	86.0	0.951	0.922	84.8

**Table 5:** Accuracy comparison of the improved ConvNeXt-U (+CBAM) model with Swin Transformer, MobileNetV3, ResUnet, and VGG16.

	Acc (%)	Precision (%)	Recall	F1	IoU (%)
ConvNeXt_U (+CBAM)	85.2	80.5	0.986	0.886	79.5
Swin Transformer	85.1	81.4	0.965	0.883	79.1
MobileNetV3	83.4	78.4	0.985	0.874	77.6
ResUnet	81.8	76.6	0.992	0.865	76.1
VGG16	80.5	75.8	0.979	0.855	74.6

**Table 6:** Comparison of model computational complexity and efficiency on an NVIDIA GeForce RTX 4070 GPU. Using float32 data format and “channel first” memory layout.

	Parameter Count	FLOPs	Throughput (Image/s)
ConvNeXt_U (+CBAM)	0.17 M	3.030 G	37
Swin Transformer	27.15 M	30.922 G	28
MobileNetV3	3.90 M	1.25 G	35
ResUnet	25.56 M	21.652 G	0.44
VGG16	138.36 M	80.673 G	0.43

## Data Availability

All code and data will be available from the authors upon request.
